# A Novel Method for Classifying Liver and Brain Tumors Using Convolutional Neural Networks, Discrete Wavelet Transform and Long Short-Term Memory Networks

**DOI:** 10.3390/s19091992

**Published:** 2019-04-28

**Authors:** Hüseyin Kutlu, Engin Avcı

**Affiliations:** 1 Computer Using Department, Besni Vocational School, Adıyaman University, Adıyaman 02300, Turkey; 2 Software Engineering Department, Technology Faculty, Fırat University, Elazığ 23000, Turkey; enginavci@firat.edu.tr

**Keywords:** classification of liver tumor, classification of brain tumor, computer-aided diagnosis, CNN, LSTM, DWT, signal classification, feature reduction, biomedical image processing

## Abstract

Rapid classification of tumors that are detected in the medical images is of great importance in the early diagnosis of the disease. In this paper, a new liver and brain tumor classification method is proposed by using the power of convolutional neural network (CNN) in feature extraction, the power of discrete wavelet transform (DWT) in signal processing, and the power of long short-term memory (LSTM) in signal classification. A CNN–DWT–LSTM method is proposed to classify the computed tomography (CT) images of livers with tumors and to classify the magnetic resonance (MR) images of brains with tumors. The proposed method classifies liver tumors images as benign or malignant and then classifies brain tumor images as meningioma, glioma, and pituitary. In the hybrid CNN–DWT–LSTM method, the feature vector of the images is obtained from pre-trained AlexNet CNN architecture. The feature vector is reduced but strengthened by applying the single-level one-dimensional discrete wavelet transform (1-D DWT), and it is classified by training with an LSTM network. Under the scope of the study, images of 56 benign and 56 malignant liver tumors that were obtained from Fırat University Research Hospital were used and a publicly available brain tumor dataset were used. The experimental results show that the proposed method had higher performance than classifiers, such as K-nearest neighbors (KNN) and support vector machine (SVM). By using the CNN–DWT–LSTM hybrid method, an accuracy rate of 99.1% was achieved in the liver tumor classification and accuracy rate of 98.6% was achieved in the brain tumor classification. We used two different datasets to demonstrate the performance of the proposed method. Performance measurements show that the proposed method has a satisfactory accuracy rate at the liver tumor and brain tumor classifying.

## 1. Introduction

Liver cancer is the fifth most common type of cancer in the world. The survival time after the diagnosis of liver cancer is about six years. Brain cancer occurs every year to between five and seven people out of 100,000 people. The survival time is between 14 months and 12 years depending on the stage of the brain cancer at the diagnosis time. Early diagnosis of tumor type is important in lengthening this period [1,2,3,4,5].

Liver and brain malignant tumors have irregular borders and they are visible intensely contrasted, radiant, and spread to surrounding tissues. Although it is easy for radiologists to determine the tumor areas, it is difficult, time consuming, and error-prone to classify the tumors as benign and malignant. Radiologists benefit from computer-assisted decision systems in order to overcome this challenge. Image processing has an important place in computer-aided diagnosis (CAD). In recent years, deep learning has overtaken conventional image processing methods. A number of studies have shown that deep learning algorithms outperform conventional methods [6,7,8]. Feature extraction is the main step in image classification with conventional image processing. This requires specialized knowledge. Eliminating the need for feature extraction in deep learning algorithms is their most prominent advantage over conventional image processing. Convolutional neural network (CNN) technology shows superior performance in image classification and pattern recognition when compared to conventional methods. As it performs quite well in image recognition, segmentation, detection, and tracking, it is widely used in image and video processing. Recurrent neural network (RNN), which is a deep learning architecture, is based on the principle of using input data as a correlated series. RNNs work like memory, keeping information across a neural network [9]. Long short-term memory (LSTM) is a special kind of RNN with a long-term memory network.

There is a spatial relationship among the pixels in the rows and columns of an image. Convolution preserves this spatial relationship while learning the features of the image. These relationships are important in classifying images. LSTM can classify the feature vectors that were obtained from CNN. Recently, studies have been done to classify images by using CNN features and the LSTM classifier. Chen et al. [10] proposed a method for image classification using features that were extracted from different CNN architectures. They proposed the use of LSTM to combine different CNN architecture features. Using the memory of LSTM, they gave feature vectors that were derived from different CNNs as input to the LSTM structure. In [11], Shahzadi et al. proposed a cascade of CNN–LSTM networks for classification of magnetic resonance (MR) brain lesion images. Feature vectors from a pretrained VGG-16 CNN model were extracted and then fed into an LSTM network to learn high-level feature representations to classify 3D brain lesion volumes into high-grade and low-grade glioma. In [12], Rachmadi et al. explored the correlations between the spatial features of the vehicle images. They attached an LSTM classifier to the end of the parallel CNNs and treated each CNN as an independent timestamp. An and Liu [13] proposed a CNN–LSTM–support vector machine (SVM) hybrid method for facial expression recognition. In [14], Guo et al. performed image classification with a CNN–RNN hybrid model. In [15], Lakhal et al. employed CNN architecture to encode remote sensing images into a feature space, and then used LSTM architecture to decode these features to predict the class labels. In [16], Zuo et al. proposed a CNN–RNN model that learns the spatial dependencies between the image regions to enhance the discriminative power of image representation.

The feature vector that was obtained from the fully connected layer of CNN represents the image. The feature vector can be considered to be a signal. Meaningful information is selected from the feature vector and the dimension of the signal is reduced when discrete wavelet transform (DWT) is applied to the CNN feature vector. This improves the performance of the classifier. Studies have used DWT to obtain the feature vector or to reduce the feature vector dimension, thus increasing the classifier performance. Liu [17] proposed a method of feature extraction and dimension reduction for high-dimensional DNA microarray data. In the study, DWT was used to characterize the localized features of the gene expression vectors. Khatami et al. [18] increased the performance of deep belief networks by using wavelet transform in radiological image classification. Yadav et al. [19] used DWT-based texture feature extraction techniques to categorize the microscopic images of the hardwood species into 75 different classes.

DWT is frequently used in microarray data analysis [20,21,22,23]. Microarray vectors are linear vectors, such as CNN feature vectors, which do not contain any clear time or space variable. In the studies, usually wavelet transform was used in gene expression analysis because of its multiresolution approach in signal processing, and the microarray data were transformed into the time-scale domain and used as classification features. Since each gene expression vector contains thousands of genes; the number of genes was viewed as the length of the signals [24].

Dimension reduction is an important strategy to improve classification performance. It has been indicated that, as the dimension increases, the sample dimension needs to exponentially increase in order to have an effective estimate of multivariate densities [25]. In this study, inspired by the above-cited studies, we obtained a CNN feature vector, and, taking advantage of DWT’s dimension reduction and feature extraction performance, we strengthened the feature vector and classified it via LSTM. After this process, we saw that DWT improved LSTM classifier performance.

The CNN–DWT–LSTM structure is proposed for image classification in this study. To our knowledge, this is the first attempt to use a CNN–DWT–LSTM structure in biomedical image processing. Under the scope of the study, images of 56 benign and 56 malignant liver tumors that were obtained from Fırat University Research Hospital were used and publicly available brain tumor datasets were used. Obtained features from CNN were classified with support vector machine (SVM) and K-nearest neighbors (KNN) classifiers. The experimental results were compared. The hybrid structure in this study is proposed as an image classification method with high classification performance.

In this study, the features of images are extracted with the pre-trained AlexNet CNN architecture. The discontinuities in the feature vectors were determined by one-dimensional (1-D) DWT that use Daubechies wavelet, reducing the size of the attribute vector. Thus, the feature vector, CNN + DWT, which is a strengthened representation of the feature vector, was formed. CNN + DWT feature vector was trained with LSTM and the tumors of liver and brain images were classified.

The rest of the study is organized, as follows. In Section 2, we review previous works that focused on handcrafted feature extraction and then present more recent work on liver and brain tumor classification. In Section 3, we give a full description of our proposed model. We provide detailed information regarding the experimental process of implementing our proposed method in Section 4. Finally, Section 5 presents the conclusions of our study.

## 2. Related Works

The classification of liver tumors images has become important in recent years. Many studies have been done with conventional image processing methods [26,27,28,29]. Many of the studies, like ours, used custom datasets. Although the accuracy rate that was obtained by our proposed method is higher than what is found in other studies, the results cannot be directly compared in terms of accuracy, because they depend on the dataset used. According to our research, there is no public dataset of human liver tumor images. Therefore, the proposed method cannot be compared with the existing methods.

Ozyurt et al. [30] used the same dataset that we used in this study. They classified raw computed tomography (CT) images with CNN and obtained a 94.6% accuracy rate. In this study, we classified the same dataset with the CNN–DWT–LSTM method and achieved a 99.1% accuracy rate. This comparison is provided in detail in the experimental results section.

The classification of brain tumors is an issue in which researchers have been working in recent years. Cheng et al. [31] focused on the classification of three types of brain tumors (i.e., meningioma, glioma, and pituitary tumor). They evaluated the efficacy of the proposed method on a large dataset with three feature extraction methods, namely, intensity histogram, gray level co-occurrence matrix (GLCM), and bag-of-words (BoW) model. When compared with using the tumor region as ROI, using the augmented tumor region as ROI improves the accuracies to 83.58%, 87.08%, and 90.59% for the intensity histogram, GLCM, and BoW model, respectively. Cheng et al. publically published the Brain Tumor data set [32,33]. Paul et al. [34] were used Cheng et al. dataset. They focused on the axial images. Two types of neural networks were used in classification: fully connected and CNN. Training neural networks over the axial data has proven to be accurate in its classifications, with an average five-fold cross validation of 91.43% on the best trained neural network. Abiwinanda et al. [35] used Cheng et al. dataset. They attempted to train a CNN to recognize the three most common types of brain tumors, i.e., the Glioma, Meningioma, and Pituitary. They implemented the simplest possible architecture of CNN; i.e., one each of convolution, max-pooling, and flattening layers, followed by a full connection from one hidden layer. They could achieve a validation accuracy of 84.19% at best.

## 3. Proposed CNN–DWT–LSTM Method

**Figure 1:**
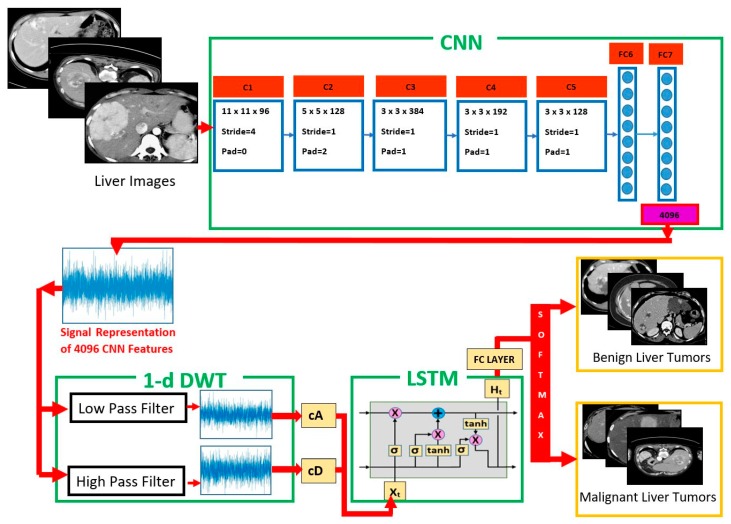
Proposed convolutional neural network (CNN)–discrete wavelet transform (DWT)–long short-term memory (LSTM) architecture for liver tumor classification.

The proposed CNN–DWT–LSTM method comprises three main parts: (i) CNN aims to obtain the feature vector of the image; (ii) DWT transforms the feature vector into a further elaborated, reduced, and strengthened signal and detect signal discontinuities; and, (iii) LSTM tries to disassemble the signal to predict a class label. Figure 1 shows a block diagram of the proposed method for liver tumor classification.

For better classification performance of LSTM, the feature vectors obtained from CNN were further elaborated, signal discontinuities detected, and their dimensions were reduced via one-dimensional (1-D) DWT. Studies in which DWT improves classifier performance by further elaborating and reducing the feature vector dimension in image processing are available in the literature [36,37,38,39,40].

In this study, the proposed method classifies liver tumors images as benign or malignant and it classifies brain tumor images as meningioma, glioma, and pituitary. The feature vectors of images that were obtained from CNN were decomposed into low-frequency components with 1-D DWT and then trained with LSTM network to classify.

### 3.1. Pre-Trained AlexNet CNN Architecture for Feature Extraction

For convolutional neural networks, in some cases there may small data to train and test the network or creating the labeled data might be expensive. Thus, transfer learning can be applied to adopt the features that have been learned in earlier settings [41]. In this study, we apply fine-tuning for AlexNet architecture that was trained with a large dataset [42].

In the process of image classification with deep learning, no preprocessing and feature extraction steps take place in conventional image classification methods. In the deep learning methods, preprocessing and feature extraction steps are performed via CNN. CNN has a layered structure. In the CNN model, the structures include convolutional layer, pooling layer, activation function [43], dropout [44], and fully connected layer [45]. The feature vectors of the image are obtained thanks to the convolutional layer. Since CNN was proposed, many effective CNN models have been designed and developed. One of these models is AlexNet architecture [46]. AlexNet architecture is used in this study, with default training values.

The idea of using CNN as a feature vector extractor was suggested in [47,48]. The results in those studies show that feature vectors as generic descriptors are a good choice for image classification tasks. The extraction of 4096 features was done from the fully connected layer before the softmax classifier of the AlexNet CNN architecture.

### 3.2. DWT as Feature Extraction and Reduction of Feature Vector Dimension

**Figure 2:**
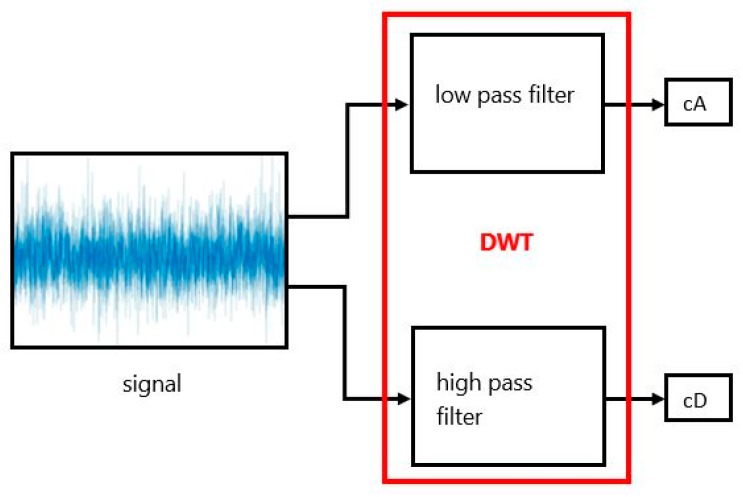
Filtering in one level of one-dimensional (1-D) DWT.

Grossmann and Morlet proposed the wavelet-transform method [49]. Wavelet analysis for CNN feature vectors can be represented as a sum of wavelets at different time shifts and scales using DWT. DWT is capable of extracting local features by separating the components of feature vectors in both time and scale. Wavelet analysis involves two elements: approximations and details. One-dimensional DWT produces two sets of coefficients: approximation coefficients (cA) and detail coefficients (cD). These coefficients are computed by convolving the signals with the low-pass filter for approximation and with the high-pass filter for detail. The convolved coefficients are downsampled by keeping the even indexed elements. cA is the most important part of the sign for many signals. The cA of the sign defines its identity. On the other hand, there is also identifying information in the cD. For instance, if the cD is removed from a human voice signal, the sound changes, but remains comprehensible. However, if cA information is removed, then the sound becomes incomprehensible. Thus, cA is used in DWT analysis. Figure 2 shows filtering in DWT.

For two-dimensional DWT, the second DWT is applied to the cA that is a component from the first DWT process and new cA and cD values are obtained. In this study, the first cA component of the signal was taken, because one-dimensional DWT was applied. Each CNN feature vector has 4096 dimensions. We performed 1-D DWT on each vector to obtain the detail coefficients. At one level, 2054 wavelet features were obtained. Thus, we improved the classifier performance by reducing the 1 × 4096 dimension feature vector to 1 × 2054. Additionally, this process further elaborated the signal. Wavelets are a family of basis functions. In this study, we used Daubechies basis 7 (db7) for wavelet analysis of the CNN feature vector. Daubechies wavelets are widely used in solving a broad range of problems, e.g., self-similarity properties of a signal or fractal problems, signal discontinuities, etc.

**Figure 3:**
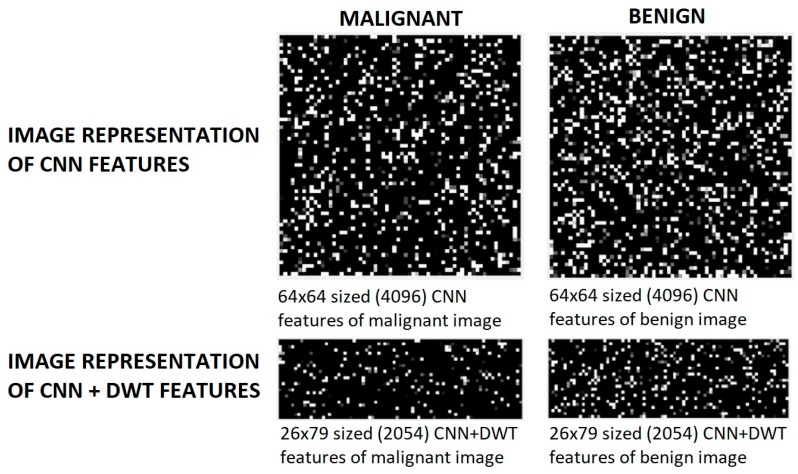
Image representation of CNN features of the liver images and image representation of cnn + dwt features that obtained from malignant and benign tumor images.

Figure 3 shows the image representation of CNN feature vector of the liver images and image representation of CNN + DWT feature vector. There are noticeable differences in the images. The differences are the discontinuity of the values in the feature vector. These discontinuities can be understood from white pixels in the rendered image of the feature vector. White pixels are more in the benign feature vectors. In addition to dimension reduction, feature selection task, DWT also detects these discontinuities.

Looking at the performance of transform based features, such as DWT, DWT performs effectively in finding continuity in object shape information. Therefore, in this study, we used DWT both as dimension reducer and to find continuity in pixel value information.

In the literature, 1-D DWT is used to extract feature vector, reduce the dimension of feature vector, and find continuity in object shape information [50,51,52]. In this study, 1-D DWT was applied to the feature vector that was obtained from CNN to increase the classification performance of LSTM. In this way, it is intended to reduce the dimension of the feature vector by preserving important features of the feature vector.

### 3.3. LSTM-Based Image Classification

CNN processes pieces of input data of a network independently from each other. While this approach is correct for uncorrelated data, it is not suitable for correlated data, such as time series. Recurrent neural networks (RNNs) emerged as a result of using correlated input data as time series.

**Figure 4:**
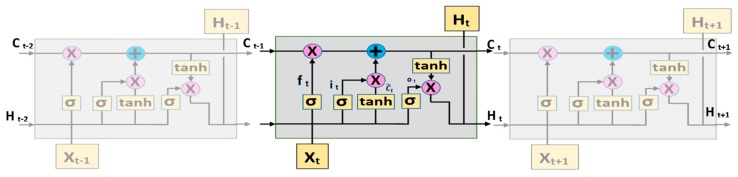
Working principle of LSTM architecture.

The LSTM architecture was proposed to solve the long-term memory problem of RNNs [53]. LSTM models were designed to prevent the long-term vanishing gradient problem. Figure 4 shows the working principle of LSTM architecture.

LSTM architecture has four gates, each of which is an artificial neural network, named input gate, forget gate, update gate, and output gate. The t-time in the mathematical expressions of these gates that are given in Equations (1)–(6) indicates the length of the input sequence. In other words, t-time is the time step of LSTM. In this study, *t*-time is the length of the DWT output.

(1) $$f_{t}=\sigma \left(W_{f}*\left[h_{t-1},x_{t}\right]+b_{f}\right)$$

The mathematical expression of the forget gate is given in Equation (1). In this equation, $f_{t}$ is the output of the memory gate at *t*-time; σ is the sigmoid function that keeps the values between 0 and 1 (normalization); $W_{f}$ is the weight value of the artificial neural network that will learn the forget; $h_{t-1}$ is the output values that are received from the previous cell; $x_{t}$ is the input values; and, $b_{f}$ is the bias weight values of the artificial neural network. At the output of the network, 1 denotes keeping the information and 0 denotes forgetting it.

(2) $$I_{t}=\sigma \left(W_{i}*\left[h_{t-1},x_{t}\right]+b_{i}\right)$$

(3) $${\tilde{c}}_{t}\;=tanh\left(W_{c}*\left[h_{t-1},x_{t}\right]+b_{c}\right)$$

In Equation (2), *I_t_* is the output of the input gate; σ is the sigmoid function; $W_{i}$ is the weight values of the artificial neural network that will learn which information should be stored in memory; $h_{t-1}$ is the output values received from the previous cell; $x_{t}$ is the input values; and, $b_{i}$ is the bias weight values of the artificial neural network. In Equation (3), ${\tilde{c}}_{t}$ is an artificial neural network output with tanh function that normalizes values between −1 and 1; $W_{t}$ is the weight values of the artificial neural network that will learn which information should be stored in memory; $h_{t-1}$ is the output values that were received from the previous cell; $x_{t}$ is the input values; and, $b_{c}$ is the bias weight values of the artificial neural network.

(4) $$C_{t}=\;C_{t-1}*\;f_{t}+\;i_{t}*\;{\tilde{c}}_{t}$$

The operators * and + in the equations mean element-wise multiplication/addition. The memory is updated in the update gate. The memory is updated with Equation (4), after the artificial neural network learns the information stored or forgotten in memory and learns candidates or newly added information with Equations (1)–(3).

(5) $$O_{t}=\sigma \left(W_{O}*\left[h_{t-1},x_{t}\right]+b_{O}\right)$$

(6) $$h_{t}=O_{t}*\tan h\left(C_{t}\right)$$

$O_{t}$ denotes the output of the output gate; $W_{o}$ is the weight values of the artificial neural network that will learn which information should be stored in memory; $h_{t-1}$ is the output values that were received from the previous cell; $x_{t}$ is the input values; and, $b_{o}$ is the bias weight values of the artificial neural network. The output value is equal to element-wise multiplication of $h_{t}$, $O_{t}$, and tanh ($C_{t}$). $O_{t}$ and $h_{t}$ are calculated with Equations (5) and (6), respectively.

The LSTM systems learn forward dependencies in sequential data. Bidirectional LSTM (BiLSTM) has been proposed to learn forward and backward dependencies in sequential data [54]. BiLSTM consists of two LSTM layers, one that operates in the forward direction and the other in the backward direction. BiLSTM structures provide successful results when sequential data are related in both the forward and backward directions.

The LSTM network consists of sequence input with one dimension, BiLSTM with 100 hidden units, 50% dropout, two fully connected layers, softmax classifier, and classification output layers. In addition, the Adam optimization method was used to train the LSTM network. The Epoch value was set to 400, mini-batch size value was set to 10, initial learn rate was set to 0.01, and gradient threshold value was set to 1.

## 4. Experimental Results

The experimental results are evaluated in the Experimental Tools, Classifier Comparisons, Performance Measurements, and Classification Performances subsections and then discussed in the Discussion section.

### 4.1. Dataset

**Figure 5:**
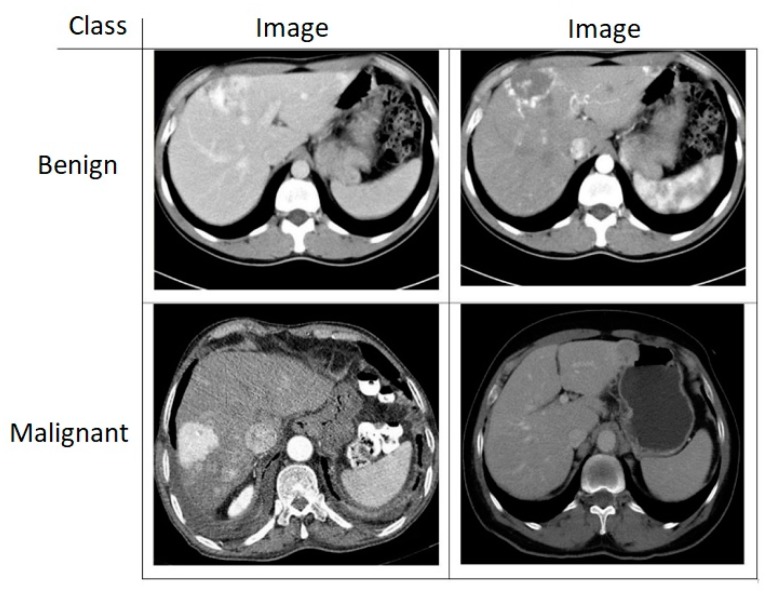
Computed tomography (CT) images of liver tumors.

In this study, CT images of benign and malignant liver tumors were classified. The malignant liver tumors in our study were composed of only hepatocellular carcinoma (HCC). Figure 5 gives the CT image samples of each tumor. The CT image dataset of liver tumors that were used in the study was obtained from the Radiology Laboratory of Fırat University Research Hospital. Each image taken to the experimental study was 663 × 650 × 3. The parameters used when acquiring images were as follows: tube voltage set to 90–130 kV; section thickness set to 1 mm slice thickness by helical CT. Algorithm evaluation was performed over the whole image without selecting the volume of interest (VOI) in the diagnosis of mass tumors. In the multiphasic CT images, the radiologist chose the section of the mass tumor with the largest area. The evaluation was made at the same level from a single incision that was obtained in different dynamic contrast phases. The dataset consists of a total of 112 images, 56 benign and 56 malignant tumors. The images were not preprocessed or resized. The required permission has not yet been received from the relevant authorities for the dataset to be made public.

**Figure 6:**
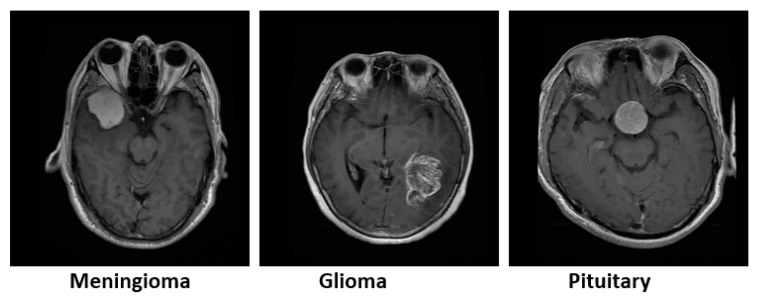
Axial brain tumor images from the public dataset provided by Jun Cheng et al.

The performance of the proposed method evaluated in a different dataset. For this purpose, a brain tumor public dataset was used. Jun Cheng et al. provided the dataset [31,32,33]. The image dataset contains 233 patients with a total of 3064 brain images with meningioma, glioma, or pituitary tumors. The images are T1-weighted contrast enhanced MRI (CE MRI) images of axial (transverse plane), coronal (frontal plane), or sagittal (lateral plane) planes. Each image contained an original size of 512 × 512 in pixels. In order to avoid confusing the convolutional neural network with three different planes of the brain that could have the same label, we focused on the axial images. Out of these images, we randomly selected 100 meningioma, 100 glioma, and 100 pituitary tumor images from different patients. Figure 6 shows the axial brain tumor images from the dataset.

### 4.2. Experimental Tools

The proposed CNN–DWT–LSTM method was run on a laptop computer with Intel Core i7 4510U processor, 8 GB RAM, and Windows 10 operating system. The codes of the application were written in MATLAB R2018a software. CNN with AlexNet architecture was used for the proposed method. A total of 4096 features were extracted from fully connected layers before the softmax layer of CNN, and then passed to SVM, KNN, and proposed CNN–DWT–LSTM classifiers. The training and test data were selected from the dataset five times in a random manner, 70% for training, and 30% for testing. Five training datasets and five testing datasets was created in this way. An equal number of images from each class was used when creating the training and test data. Classifiers were run five times for five datasets. The tables provide the mean and standard deviation of results.

### 4.3. Classifier Comparisons

The performance that was achieved by the proposed method was compared to that of k-nearest neighbors (KNN) and support vector machine (SVM) [55,56]. The classifiers were trained with a 1 × 4096 vector that was obtained from CNN.

### 4.4. Performance Measurements

Validity evaluation of this study was performed with sensitivity, precision, accuracy, and Youden’s index scales, as assessed with true positive (TP), true negative (TN), false positive (FP), and false negative (FN) values [57]. Equations (7)–(10) provide the mathematical equations of the scales:

(7) $$\mathrm{Accuracy}\;=\;\frac{\left(\mathrm{TP}\;+\;\mathrm{TN}\right)}{\left(\mathrm{TP}\;+\;\mathrm{TN}\right)\;+\;\left(\mathrm{FP}\;+\;\mathrm{FN}\right)}$$

(8) $$\mathrm{Sensitivity}\;=\;\frac{\left(\mathrm{TP}\right)}{\left(\mathrm{TP}\;+\;\mathrm{FN}\right)}$$

(9) $$\mathrm{Specificity}\;=\;\frac{\left(\mathrm{TN}\right)}{\left(\mathrm{TN}\;+\;\mathrm{FP}\right)}$$

Youden index (J) = Sensitivity + Specificity − 1(10)

where TP indicates the number of images classified as malignant that were malignant; TN indicates the number of images classified as benign that were benign; FP indicates the number of images classified as malignant but were benign; and, FN indicates the number of images that were classified as benign but were malignant.

Table 1 provides the performance values of classification of the 1 × 4096 feature vector of liver tumors obtained from CNN using baseline CNN.

According to Table 1, classification with baseline CNN has a 93.8% accuracy rate. Table 2 provides the performance values of classification of the 1 × 4096 feature vector of liver tumors that were obtained from CNN using SVM, KNN, and LSTM classifiers.

According to Table 2, classification with LSTM is greater than SVM (93.8%) and KNN (90.2%), with an accuracy rate of 95.4%. In order to improve the performance, the 1 × 4096 feature vector that was obtained from CNN was decomposed with 1-D DWT.

The 1 × 2054 feature vector that was obtained from 1-D DWT was reclassified with classifiers. Table 3 shows the results.

Table 3 shows the results of the proposed method. One-dimensional DWT, which is a widely used method for improving the performance of signal classification applications, was applied to the 1 × 4096 feature vector. 1 × 2054 approximation coefficients (cA) vector and a 1 × 2054 detail coefficients (dA) vector were obtained as a result of this process. The approximation coefficients vector (cA) was given as input to the LSTM structure and KNN and SVM classifiers. Table 3. provides the results of the classification process. With an accuracy rate of 99.1%, the proposed CNN–DWT–LSTM method had better performance than the KNN (96.4) and SVM (98.2%) classifiers.

Different wavelet transform methods, the discrete cosine transform (DCT) and fast Walsh–Hadamard transform (FWHT), were used with the proposed method to demonstrate that wavelet transform improves the performance of LSTM. The results were compared with DWT and they are shown in Table 4.

As can be seen in Table 4, the DCT and FWHT wavelet transform methods, including DWT, improved the classification performance of LSTM. DCT and FWHT did not reduce the feature vector dimension when performing wavelet transform. DWT, in addition to the wavelet transform process, reduced the dimension of the feature vector and gave a better result than DCT and FWHT.

Although the accuracy of the proposed CNN + DWT + LSTM classifier is higher than the methods that were used in other studies, it is not correct to compare the classification performance, because different databases were used in those studies. According to our research, there is no public database for the classification of liver tumors. Ozyurt et al. [30] used the same database as this study. Table 5 provides a comparison of the CNN + DWT + LSTM classifier and the baseline CNN method in [30].

Table 5 shows that the proposed method increased the classification success. The main contribution of this study consists of a postprocessing step, whereby CNN-generated features are further elaborated and signal discontinuities detected and reduced before being passed on to a classifier. We used DWT before the feature vector was passed on to a classifier, and Table 3 shows that this step increased the accuracy. Table 4 shows that other discrete transform methods (DCT, FWHT) can increase the accuracy of the classifier performance.

### 4.5. Classification Performance

**Figure 7:**
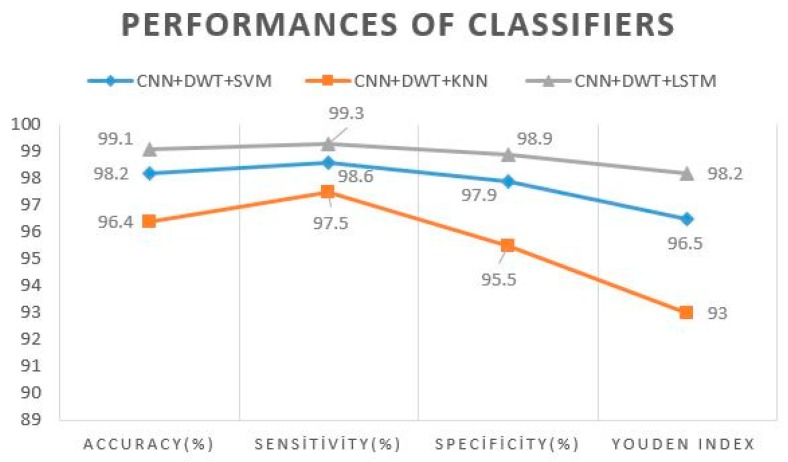
Performance of classifiers.

Figure 7 shows that the proposed classifier had the best performance, with 99.1% accuracy, 99.3% sensitivity, and 98.9% specificity.

**Figure 8:**
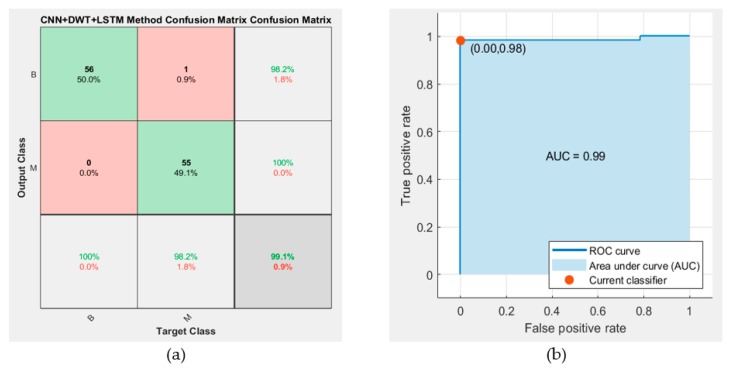
(**a**) Confusion matrix of proposed method; (**b**) Receiver operating characteristic (ROC) curve analysis of proposed method.

Figure 8 shows the confusion matrix and the receiver operating characteristic (ROC) curve analysis of the proposed CNN–DWT–LSTM method.

50% dropout was applied to the LSTM network to prevent overfitting. When the dropout amount was increased, there was a consistent decrease in the classification performance of the testing and training data. The results show that the network has no overfitting problem.

**Figure 9:**
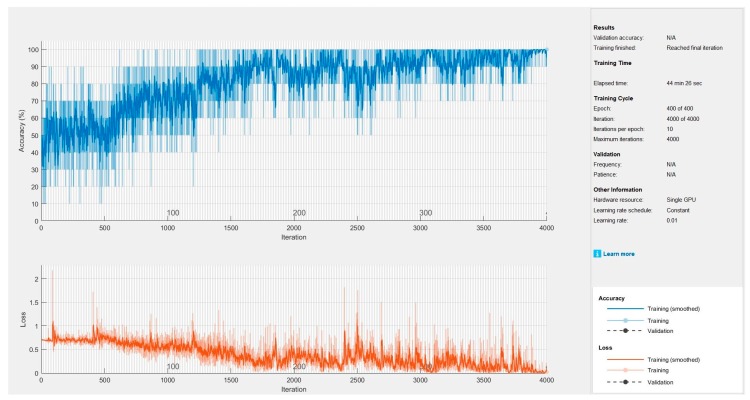
Training progress of proposed methods.

Figure 9 shows the plotting of a training curve (loss curve) of the network during the training phase. As shown in the figure, the training time of the proposed network is about 45 min, the iteration value is 4000, and the learning rate value is 0.01.

The liver data set that was used in this study and the public brain tumor data set were evaluated under the same conditions. The experimental method with fivefold cross-validation testing was employed. In the fivefold cross-validation testing protocol, the training data was divided into five subsets and the processing was repeated five times. The results were averaged and are shown in Table 6.

The performance measurement values in Table 6 show the classification performance of the proposed method in a different database. Implementing DWT to CNN attributes increased the performance of the classifiers. In the same data set, Cheng et al. [31,32,33] obtained 71.39% classification accuracy and Paul et al. [34] obtained 91.43% classification accuracy with the CNN architecture that they designed. Abiwinanda et al. [35] obtained a classification accuracy rate of 84.19% with the CNN architecture that they designed.

### 4.6. Discussion

We can explain the proposed method, as follows. The first stage is to apply a CNN that has AlexNet architecture to an image. The output of this stage is a 1 × 4096 feature vector of the image. One-dimensional DWT is the second stage. The input of the second stage is the 1 × 4096 feature vector and the output is a 1 × 2054 approximation coefficients (cA) vector. The final stage is the LSTM structure. The input of this stage is the 1 × 2054 approximation coefficients (cA) vector, and the output is the prediction of the class of the image.

The proposed method was compared with SVM and KNN classifiers under the same conditions in terms of performance. Classification accuracy, sensitivity, precision, and Youden’s index parameters were used to measure the performance of the CNN–DWT–LSTM method. Table 3 shows the classification comparisons. These results prove the superiority of the CNN–DWT–LSTM method.

This study was conducted by targeting the following items:

- Taking advantage of CNN’s success in feature extraction and obtaining a feature vector of 1 × 4096.
- Considering the 1 × 4096 feature vector as a signal and separating the signal into low-frequency components by utilizing the success of 1-D DWT in dimension reduction, feature extraction, and detect signal discontinuities.
- Taking advantage of the success of the LSTM structure in signal classification and obtaining a new robust image classifier.
- Experimental results show that the study reached its goals.

## 5. Conclusions

In this study, we proposed a new method that obtains the features of images of the liver and brain through CNN, analyzes the features through 1-D DWT, and learns and classifies the features through LSTM. The experimental results show that the CNN–DWT–LSTM method attains an accuracy of 99.1% and it is ahead of powerful classifiers, such as KNN and SVM. This study also illustrates the usability of CNN for the purpose of feature extraction, of DWT for the purpose of signal analysis (CNN feature vector), and of LSTM for the purpose of signal classification. This study shows that applying DWT to the CNN feature vector can increase the performance of the classifier and that analyzing the feature vector of CNN as a signal can extract important information that improves clasifier performance. It shows that discrete transform methods can be used for this process. In experimental studies, it was observed that applying different wavelet transform methods to the feature vector yielded successful results. With the experience that we share in this study, we are planning to conduct new studies by applying statistical or different signal processing methods to the CNN feature vector for image classification, object detection, and problem tracking.

## Figures and Tables

**Table 1:** Baseline CNN classification.

Method	Classifier	Accuracy (%)	Sensitivity (%)	Specificity (%)	Youden’s Index
CNN	Softmax	93.8 ± 0.8	94 ± 1.4	93.6 ± 1.6	0.87 ± 0.01

**Table 2:** Classification of 1 × 4096 feature vector obtained from CNN with support vector machine (SVM), K-nearest neighbors (KNN), and LSTM.

Method	Accuracy (%)	Sensitivity (%)	Specificity (%)	Youden’s Index
CNN + SVM	93.8 ± 0.6	93.6 ± 1.0	93.9 ± 1.5	0.87 ± 0.01
CNN + KNN	90.2 ± 0.6	90.7 ± 1.4	89.8 ± 2.0	0.80 ± 0.01
**CNN + LSTM**	**95.4 ± 1.2**	**95.0 ± 0.8**	**95.7 ± 1.6**	**0.90 ± 0.02**

**Table 3:** Classification of 1 × 4096 feature vector of liver tumors obtained from CNN with SVM, KNN, and LSTM.

Method	Accuracy (%)	Sensitivity (%)	Specificity (%)	Youden’s Index
CNN + DWT + SVM	98.2 ± 1.4	98.6 ± 0.8	97.9 ± 2.3	0.96 ± 0.02
CNN + DWT + KNN	96.4 ± 0.6	97.5 ± 1.0	95.5 ± 0.9	0.93 ± 0.01
**CNN + DWT + LSTM**	**99.1 ± 0.9**	**99.3 ± 1.0**	**98.9 ± 1.0**	**0.98 ± 0.01**

**Table 4:** Application of different discrete transform methods in the proposed method.

Method	Accuracy (%)	Sensitivity (%)	Specificity (%)	Youden’s Index
CNN + DCT + LSTM	98.2 ± 1.1	98.2 ± 1.3	98.2 ± 1.3	0.96 ± 0.02
CNN + FWHT + LSTM	97.3 ± 0.6	97.2 ± 0.9	97.5 ± 1.0	0.94 ± 0.01
**CNN + DWT + LSTM**	**99.1 ± 0.9**	**99.3 ± 1.0**	**98.9 ± 1.0**	**0.98 ± 0.01**

**Table 5:** Comparison with the study [30] that used the same database as this study.

Image Size	Method	Accuracy (%)	Sensitivity (%)	Specificity (%)	Youden’s Index
Raw CT Images	CNN [30] Softmax	94.6	92.8	96.4	0.89
**Raw CT Images**	**CNN + DWT + LSTM**	**99.1**	**99.3**	**98.9**	**0.98**

**Table 6:** Classification performance of CNN features of brain tumor images.

Method	Accuracy (%)	Sensitivity (%)	Specificity (%)	Youden’s Index
CNN + KNN	83.6 ± 0.01	83.6 ± 0.01	91.8 ± 0.005	0.75 ± 0.01
CNN + SVM	87.3 ± 0.01	87.3 ± 0.01	93.6 ± 0.008	0.81 ± 0.02
CNN + LSTM	87.5 ± 0.01	87.5 ± 0.01	93.7 ± 0.007	0.81 ± 0.02
CNN + DWT + KNN	85.91 ± 0.02	85.91 ± 0.02	92.95 ± 0.01	0.78 ± 0.03
CNN + DWT + SVM	92.09 ± 0.008	92.08 ± 0.008	96.04 ± 0.004	0.88 ± 0.01
**CNN + DWT + LSTM**	**98.66 ± 0.01**	**98.66 ± 0.01**	**99.33 ± 0.008**	**0.98 ± 0.02**
